# Supplementary material for: Exploring Alcohol-Related Behaviours in an Adult Sample with Anorexia Nervosa and Those in Recovery
Source: Nutrients. 2024 Mar 4;16(5):732. doi: 10.3390/nu16050732 (PMC10934192; doi:10.3390/nu16050732)
Supplement: Supplementary file 1 [file nutrients-16-00732-s001.zip › nutrients-2846439-supplementary.pdf]

Table S1. Descriptive statistics of AUDIT scores with the statistical significance of the group comparison from the ANCOVA models for Caucasian participants divided into three groups: AN, recovered AN, and healthy controls.

Variables	Anorexia Nervosa (n = 52)	Recovered AN (n = 23)	Healthy Controls (n = 30)	Group Comparisons			
	M, SD	*M, SD*	*M, SD*	**Total Model**	**AN vs. HC**	**AN vs Rec-AN**	**HC vs Rec-AN**
Alcohol Consumption	2.88 (2.87)	4.57 (2.71)	4.47 (1.63)	F (2) = 7.093 $p < 0.001$	p = 0.002*	p = 0.005*	p = 0.928
Alcohol Dependence	0.48 (1.87)	0.65 (1.11)	0.33 (0.60)	F (2) = 2.326 $p = 0.103$	p = 0.314	p = 0.278	p = 0.035
Alcohol-Related Problems	1.34 (2.97)	1.66 (1.92)	0.57 (0.82)	F (2) = 5.058 p = 0.042*	p = 0.452	p = 0.015*	p = 0.043*
AUDIT Total	4.71 (6.48)	6.87 (4.79)	5.34 (2.48)	F (2) = 3.255 p = 0.040*	p = 0.026*	p = 0.004*	p = 0.410

Note: This table illustrates the descriptive statistics and group comparisons for the scores of the Alcohol Use Identification Test (AUDIT) across three groups in the Caucasian sample. *M* and *SD* represent mean and standard deviation, respectively. * indicates significance at the 0.05 level.
